# Contralesional Structural Plasticity in Different Molecular Pathologic Subtypes of Insular Glioma

**DOI:** 10.3389/fneur.2021.636573

**Published:** 2021-04-14

**Authors:** Zhenxing Huang, Gen Li, Zhenye Li, Shengjun Sun, Yazhuo Zhang, Zonggang Hou, Jian Xie

**Affiliations:** ^1^Department of Neurosurgery, Beijing Tiantan Hospital, Capital Medical University, Beijing, China; ^2^China National Clinical Research Centre for Neurological Diseases, Beijing, China; ^3^Neuroimaging Center, Beijing Neurosurgical Institute, Capital Medical University, Beijing, China; ^4^Beijing Neurosurgical Institute, Capital Medical University, Beijing, China

**Keywords:** insular glioma, neuroplasticity, brain structural plasticity, molecular pathology, VBM

## Abstract

Neuroplasticity may preserve neurologic function in insular glioma, thereby improving prognosis following resection. However, the anatomic and molecular bases of this phenomenon are not known. To address this gap in knowledge, the present study investigated contralesional compensation in different molecular pathologic subtypes of insular glioma by high-resolution three-dimensional T1-weighted structural magnetic resonance imaging. A total of 52 patients with insular glioma were examined. We compared the gray matter volume (GMV) of the contralesional insula according to histological grade [low-grade glioma (LGG) and high-grade glioma (HGG)] and molecular pathology status [isocitrate dehydrogenase (IDH) mutation, telomerase reverse-transcriptase (*TERT*) promoter mutation, and 1p19q codeletion] by voxel-based morphometry (VBM). A cluster of 320 voxels in contralesional insula with higher GMV was observed in glioma with IDH mutation as compared to IDH wild-type tumors by region of interest-based VBM analysis (family-wise error-corrected at *p* < 0.05). The GMV of the entire contralesional insula was also larger in insular glioma patients with IDH mutation than in patients with wild-type IDH. However, there was no association between histological grade, *TERT* promoter mutation, or 1p19q codeletion and GMV in the contralesional insula. Thus, IDH mutation is associated with greater structural compensation in insular glioma. These findings may be useful for predicting neurocognitive and functional outcomes in patients undergoing resection surgery.

## Introduction

Neuroplasticity occurs across the human life span. Developmental and adaptive plasticity underlie experience-related changes resulting from modification of the environment, physical exercise, or cognitive training (1). Additionally, reactive functional and structural plasticity is responsible for restoring normal brain function following injury, a process known as cortical remodeling or reorganization (2). Neuroimaging studies have revealed that this process occurs in both the lesioned and intact hemispheres in stroke, brain trauma, and glioma (3–10).

The pattern of cortical remodeling in glioma has been reported to be hierarchical (6). The compensation could be launched firstly within the lesioned area and then enlarged to the peritumor area. If it was still insufficient, the ipsilateral and even the contralateral hemispheres could be recruited to the remodeling process. And this hierarchical pattern is especially suited for low-grade gliomas (LGGs), because they exhibited a slow-growing, less-invasive feature, which left enough time for this remodeling process. As it happens, the insular cortex is a common location for LGGs (11, 12); it is also involved in multiple brain functions, acting as a hub for neural circuits involved in language processing, emotion, cognitive control, and decision making (13). Most insular glioma patients are diagnosed following seizure or headache or during routine physical examination, and tumor resection typically has an acceptable neurologic outcome (14, 15), implying that neuroplastic changes occur during gliomagenesis. Along this perspective, Almairac et al. (7) observed structural remodeling in unilateral low-grade insular glioma patients, whereby gray matter volume (GMV) of the contralesional insula was increased relative to healthy control subjects. It was speculated that the slow rate of growth and low invasiveness of low-grade insular glioma suited that hierarchical remodeling pattern well.

It should be noted that the 2016 World Health Organization (WHO) classification for glioma added the molecular subtype [isocitrate dehydrogenase (IDH) or telomerase reverse-transcriptase promoter (TERTp) mutation and 1p19q codeletion], which is highly associated with tumor invasiveness and prognosis (16, 17). There were even evidence that the IDH wild-type LGGs should be treated as glioblastomas (GBMs, Grade IV), as they shared similar clinical and genetic characteristics (18). Therefore, except classical histological grading, different molecular subtypes might also reflect different biological behaviors and might lead to different neuroplastic results. However, most studies investigating neuroplasticity in glioma have compared patients and healthy control subjects, without examining differences that exist according to the histological grade or molecular pathologic subtype of glioma.

Therefore, we designed the present study to investigate contralesional compensation in different histological grades, especially molecular pathologic subtypes of insular glioma by high-resolution three-dimensional (3D) T1-weighted (T1W) structural magnetic resonance imaging (MRI) and voxel-based morphometry (VBM) analysis.

## Materials and Methods

### Participants and Grouping

A total of 52 patients diagnosed with unilateral insular glioma at Beijing Tiantan Hospital were enrolled in this study. Detailed information on molecular pathology including 6-*O*-methylguanine (O6-MeG)-DNA methyltransferase (MGMT) promoter methylation status, IDH mutation, TERTp mutation, and 1p19q codeletion was available for all patients. MGMT promoter methylation has been shown to inhibit apoptosis and increase sensitivity to temozolomide (19, 20), but as it has little clinical significance in gliomagenesis, it was not investigated in this study.

The patients were grouped as IDH mutation (IDH-mu) and IDH wild type (IDH-wt), TERTp-mu and TERTp-wt, and 1p19q codeletion (1p19q-codel) and non-1p19q-codel. Additionally, we also involved histological pathology, where patients were grouped as LGG (WHO II) and high-grade glioma (WHO III and IV, HGG). The study was approved by the Institutional Review Board of the Beijing Tiantan Hospital. All the participants signed a written informed consent form.

The number of patients was relatively small when they were further grouped according to the side of the brain in which the lesion was located (left vs. right). Therefore, in order to enhance statistical power, all left-tumor MRIs were flipped along the X axis in the FMRIB Software Library (FSL) before preprocessing. Then the right and left insula could be consistently referred to as the “lesioned insula” and “contralesional insula,” respectively. Statistical analyses were performed with the flipped images and focused on the contralesional insula. However, this flipping operation could introduce bias because of the asymmetry between certain brain regions in the left and right hemispheres (21). In order to exclude this possibility, we compared the contralesional insula between the right- and left-sided tumors; as we did not observe any differences, we applied the flipping operation to our dataset.

Aging can affect GMV; hence, studies comparing patients and healthy participants typically consider age as an uninteresting variable (3, 7, 22). It should be noted that HGG, IDH-wt, and TERTp-mu in glioma were found to be closely related to older age (16, 23), which was also the case in our study (Table 1). Therefore, setting patient age as a covariate in our analyses could eliminate the effects of different molecular pathologic subtypes on GMV, which we should avoid in the present study. On the other hand, recent studies have reported that the decline in insular volume slows or even stops during aging (24, 25), suggesting that age has little effect on GMV of the insular cortex. Nonetheless, given that GMV starts to decrease at the age of 40 years (26, 27), which was also the median age in our cohort, we compared GMV of the contralesional insula between patients aged ≥40 years and those aged <40 years. As no difference was found, we considered that age did not affect GMV of the contralesional insula, and it was not included as a covariate in the statistical analysis.

**Table 1:** Demographic characteristic of different grades and molecular pathology statuses.

**Variables**	**Grade and molecular pathology status**											
	**IDH-mu**	**IDH-wt**	***P***	**TERT-mu**	**TERT-wt**	***P***	**1p19q-codel**	**Non-1p19q-codel**	***P***	**LGG**	**HGG**	***P***
Total no.	38	14	NA	21	31	NA	14	38	NA	26	26	–
Gender, M/F, *n*	23/15	8/6	0.83	13/8	18/13	0.78	7/7	24/14	0.39	16/10	15/11	0.778
Age (Mean ± SD), years	38.84 ± 9.94	53.57 ± 12.48	<0.001	46.95 ± 10.69	40 ± 12.93	0.047	41.29 ± 11.32	43.37 ± 12.94	0.6	37.04 ± 0.51	48.58 ± 12.50	<0.001
TV (Mean ± SD), ml	54.15 ± 42.65	57.58 ± 24.74	0.78	51.95 ± 32.84	57.19 ± 42.22	0.63	45.99 ± 28.27	58.42 ± 41.4	0.31	41.81 ± 32.13	68.34 ± 40.22	0.011
Scanner type, P/S, *n*	4/34	4/10	NA	2/19	6/25	NA	1/13	7/31	NA	3/23	5/21	NA
Grade II (*n* = 26)	26	0	NA	11	15	NA	11	15	NA	–	–	–
Grade III (*n* = 16)	9	7	NA	7	9	NA	3	13	NA	–	–	–
Grade IV (*n* = 10)	3	7	NA	3	7	NA	0	10	NA	–	–	–

*SD, Standard deviation; TV, Tumor volume; P, Philips Ingenia 3.0T scanner; S, Siemens Prisma 3.0T scanner; NA, Not applicable*.

### MRI

3D T1W structural images were acquired at our center with two different scanners: the Ingenia 3.0T (Philips, Amsterdam, The Netherlands) (P) and Prisma 3.0T (Siemens, Munich, Germany) (S). The acquisition program was as follows: resolution = 1 × 1 × 1 mm, field of view = 256 × 256 mm, slice thickness = 1 mm, flip angle = 8°, repetition time = 6.49/1.56 s, and echo time = 3.042/1.56 ms (for P/S). In addition, conventional magnetic resonance (MR) sequences including T2-weighted (T2W), fluid-attenuated inversion recovery (FLAIR) and T1W images with intravenous injection of a gadolinium contrast agent were routinely acquired.

### Lesion Tracing

In order to visualize the lesion in the insula and calculate tumor volume (TV), the tumor mask was delineated in T2W or FLAIR images by a neurosurgeon with 8 years of experience using MRIcron (https://www.mccauslandcenter.sc.edu/crnl/tools). Lesion masks and corresponding images for tracing were normalized to standard Montreal Neurological Institute (MNI) space using SPM12 (https://www.fil.ion.ucl.ac.uk/spm/software/spm12/). A reconfirmation procedure was performed by the neurosurgeon after normalization for more accurate matching to the original lesion range. The normalized and rechecked lesion masks were used for lesion overlap with MRIcroGL (https://www.mccauslandcenter.sc.edu/mricrogl).

### Image Preprocessing

Structural image preprocessing was performed with SPM12 and the CAT12 toolbox in MATLAB. The images were manually reoriented to the anterior commissure, which was defined as the origin (mm coordinates: 0, 0, 0). The “Segment Data” module of CAT12 was used to segment the structural images into the gray matter (GM), white matter, and cerebrospinal fluid. In this process, the original structural images were normalized to MNI-152 standard space with an isotropic voxel resolution of 1.5 × 1.5 × 1.5 mm, and we also modulated the spatial normalized data in order to maintain its original GMV. We checked the data quality to verify the segmentation and normalization results. Total intracranial volume (TIV) was determined using the “Estimate TIV” module. The modulated GM maps of each participant were smoothed with an 8-mm full-width at half-maximum Gaussian kernel. VBM analyses were performed on the smoothed images generated during the last preprocessing step.

### Statistical Analysis

VBM analysis was applied to imaging data. Based on the general linear model, the two-sample *t*-test was used for three types of comparisons: (1) left-sided vs. right-sided tumor patients to exclude the effect of brain structural asymmetry; (2) patients aged ≥40 vs. <40 years to exclude the effect of aging; and (3) LGG vs. HGG, IDH-mu vs. IDH-wt, TERTp-mu vs. TERTp-wt, and 1p19q-codel vs. non-1p19q-codel to evaluate the effect of molecular pathology on contralesional compensation in insular glioma.

As we focused only on alterations in the contralesional (left) insular cortex, we generated a left insular mask as the region of interest (ROI) using the WFU_PickAtlas toolbox (https://www.nitrc.org/projects/wfu_pickatlas/) based on the automated anatomical labeling template. We used this mask in the three abovementioned comparisons. The ROI-based analysis highlights the changing patterns of certain brain areas and provides enhanced statistical control (7). Patient sex, TIV, TV, and MR scanner type were covariates in all of the comparisons. An absolute masking threshold of 0.2 was also set in all comparisons (28). A voxel-level family-wise error (FWE) correction at *P* < 0.05 with a spatial extent threshold of 50 voxels was regarded as significant. For comparisons that passed the FWE correction in VBM, we further compared the GMV of significant clusters and the entire insula. Non-imaging data for continuous and categorical variables were analyzed with the two-sample *t*-test and chi-squared test, respectively. A *P* < 0.05 in a two-tailed test was considered statistically significant.

## Results

### Clinical and Demographic Characteristic of Study Population

**Figure 1:**
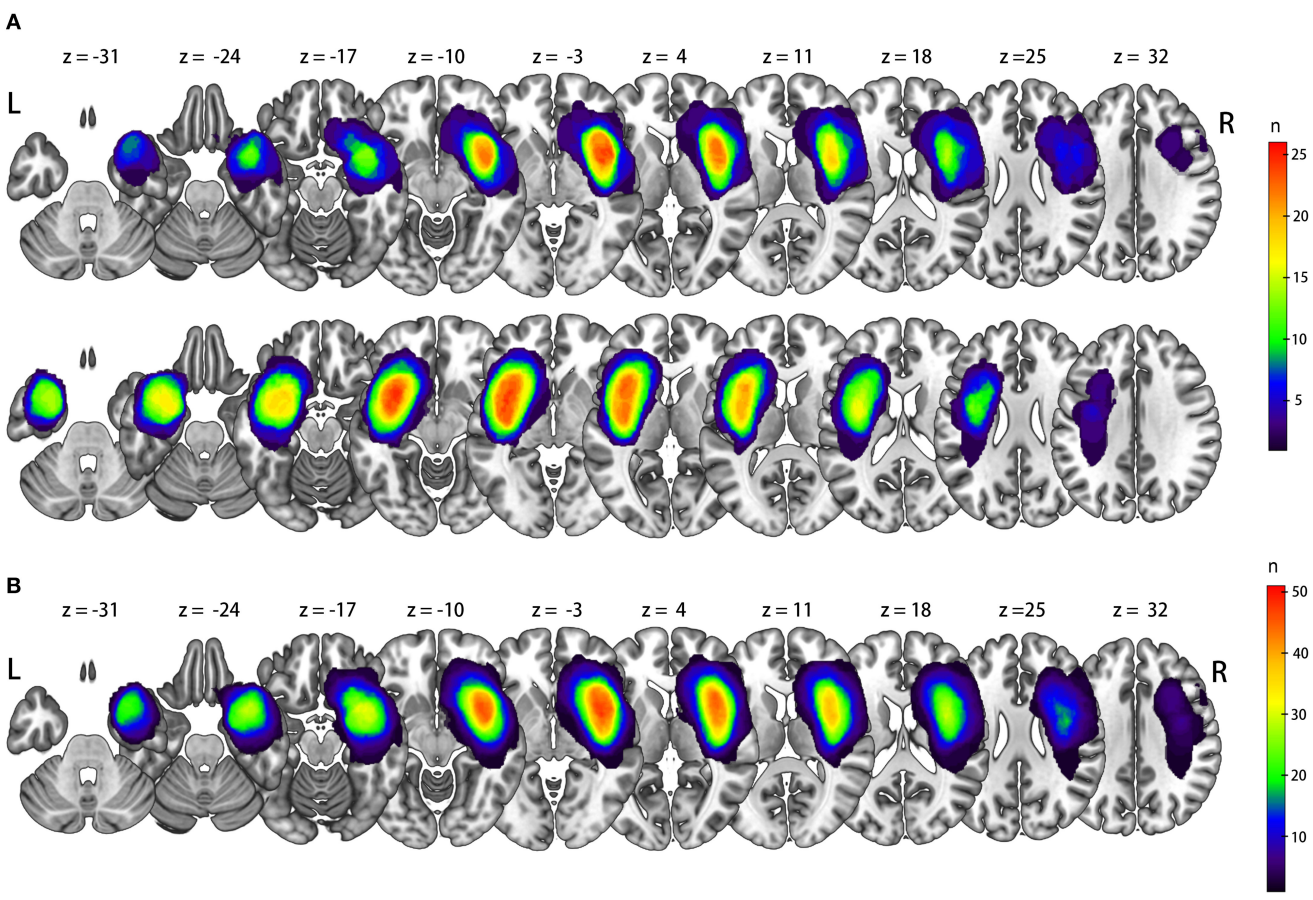
Lesion overlap for original side **(A)** and all left tumor flipped via X axis **(B)**.

In 52 patients with insular glioma (Grade II, *n* = 26; Grade III, *n* = 16; Grade IV, *n* = 10; male, *n* = 31; female, *n* = 21; median age, 40 years old), 26 tumors were located in the left insula, and 26 were located in the right insula. The lesion overlap is shown in Figure 1A. We flipped the image of left-sided tumors so that in all 52 patients, the tumor was in the right insula, and the left side was contralesional (Figure 1B). Detailed information on the molecular pathology of the tumors is shown in Table 1, and detailed demographic data are shown in Table 2.

**Table 2:** Demographic characteristic of different tumor sides and age.

**Variables**	**Left tumor**	**Right tumor**	***P***	**Age ≥40 years**	**Age <40 years**	***P***
Total No.	26	26	NA	28	24	NA
Gender, M/F, *n*	15/11	16/10	0.778	18/10	13/11	0.459
Age (Mean ± SD), years	44.42 ± 13.76	41.19 ± 11.02	0.354	51.89 ± 8.84	32.21 ± 5.85	<0.001
TV (Mean ± SD), ml	65.21 ± 37.12	44.93 ± 37.74	0.056	53.88 ± 30.75	56.46 ± 46.52	0.812
Scanner type, P/S, *n*	8/18	0/26	NA	4/24	4/20	NA

*SD, Standard deviation; TV, Tumor volume; P, Philips Ingenia 3.0T scanner; S, Siemens Prisma 3.0T scanner; NA, Not applicable*.

### ROI-Based VBM Analysis

We examined whether the GMV of the contralesional insula differed between patients with left and right insular gliomas. The results of this comparison showed that no voxels survived the FWE correction. We next compared patients aged ≥40 and <40 years to determine whether aging affected the GMV of the contralesional insula. This comparison also did not reveal significant voxels. Thus, tumor side and patient age were unrelated to the GMV of contralesional insula in our cohort.

Next, we first compared the GMV between LGG and HGG, and the results showed that no voxel survived the FWE correction. And the GMV in the entire contralesional insular cortex was also indifferent (**Figure 3** and **Table 4**).

**Figure 2:**
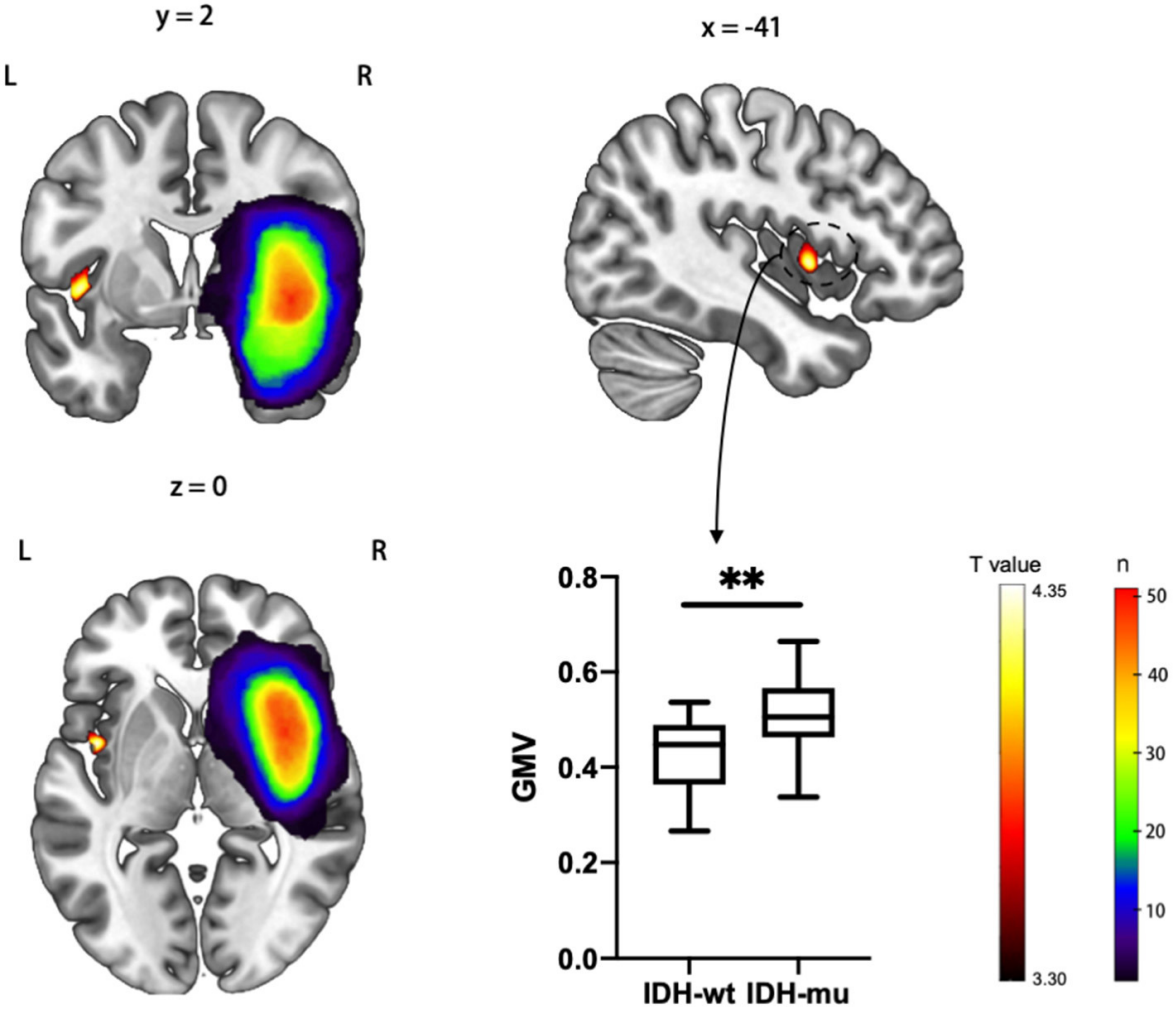
*T* statistical parametrical map of IDH-mu > IDH-wt in ROI (left insula) based VBM analysis. The box plot showed the GMV of significant clusters between IDH-wt and IDH-mu insular glioma. **P < 0.01.

**Figure 3:**
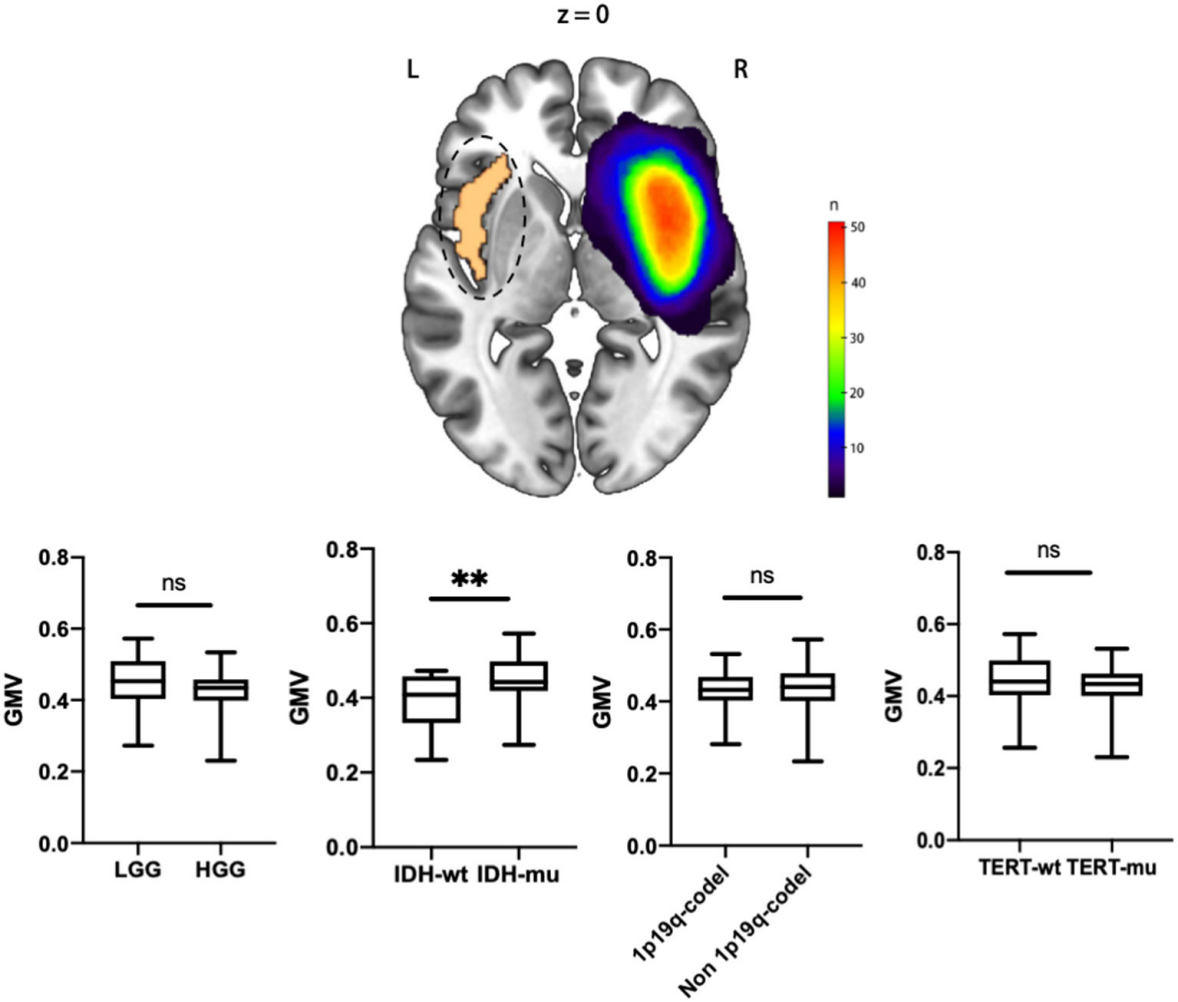
The entire contralesional insula GMV comparisons between different histological grade and molecular pathology status. **P < 0.01 ns, for not significant.

Then, we investigated whether molecular pathology in glioma is associated with the observed contralesional structural reorganization. A cluster of 320 voxels located in both anterior and posterior insular cortexes showed a significantly increased GMV in IDH-mu relative to IDH-wt (Figure 2 and Table 3). We extracted the GMV value of the significant cluster and the entire insula and found that they were both significantly higher in IDH-mu than in IDH-wt (Figure 3 and Table 4).

**Table 3:** VBM analysis for IDH-mu > IDH-wt.

**Cluster size (voxels)**	***P*-value**	**Peak MNI coordinates**			***t* score peak level**	**Anatomy location**
		***x***	***y***	***z***		
320	0.003	−44	2	0	4.39	Anterior insular cortex
		−39	−9	15	3.58	Posterior insular cortex

**Table 4:** *t*-test for GMV in different groups.

**Variables**	**GMV (mean** **±** **SD)**											
	**LGG**	**HGG**	***P***	**IDH-mu**	**IDH-wt**	***P***	**TERT-mu**	**TERT-wt**	***P***	**1p19q-codel**	**Non-1p19q-codel**	***P***
Active region	–	–	–	0.511 ± 0.075	0.427 ± 0.086	0.0011	–	–	–	–	–	–
Entire contralesional insular cortex	0.449 ± 0.074	0.415 ± 0.071	0.096	0.448 ± 0.064	0.390 ± 0.082	0.0096	0.421 ± 0.067	0.440 ± 0.078	0.377	0.431 ± 0.062	0.433 ± 0.078	0.909

However, no voxel survived in the comparison of TERTp-mu vs. TERTp-wt and 1p19q-codel vs. non-1p19q-codel. Moreover, we did not observe any GMV differences in the entire contralesional cortex in the comparisons (Figure 3 and Table 4).

## Discussion

The results of this study demonstrated for the first time that IDH mutation in insular glioma leads to morphologic compensation in the contralesional insula. TERTp mutation and 1p19q codeletion did not have this effect. The reorganization in the contralesional hemisphere could be the result of disinhibition (29), which has been observed in stroke patients on a functional level (30–32). However, glioma is a progressive disease that causes increasing damage over time. Structural reorganization with tumor growth has been observed not only in the region adjacent to the lesion but also in the contralateral hemisphere (6). Contralesional plasticity was shown to be positively correlated with the degree of impairment (29). Under these circumstances, functional plasticity of existing synapses—which depends on synaptic efficacy (33)—may not be sufficient to restore neuronal activity when structural plasticity involving synaptogenesis and axonal remodeling are required (34–36). Thus, structural remodeling of the contralesional hemisphere in insular glioma patients may depend on the duration between the onset of the lesion and detection and the extent of damage within this duration.

Along with this perspective, we could infer that insular glioma patients with IDH mutation had a longer disease course than those with wild-type IDH and accumulate damage to the insula slowly. In fact, IDH mutation has also been reported to be an early event in gliomagenesis that precedes the occurrence of TERTp mutation and 1p19q codeletion (37), which might be the reason for the absence of contralesional reorganization in the latter two genetic alterations. IDH catalyzes the conversion of isocitrate into α-ketoglutarate (α-KG), whereas the enzyme produced by the mutated IDH gene further converts α-KG into 2-hydroxyglutarate (α-HG). Excess α-HG inhibits α-KG-dependent enzymes and causes alterations in cellular metabolism, epigenetic regulation, redox state, and DNA repair, all of which contribute to carcinogenesis including acute myeloid leukemia (AML), chondrosarcoma, and glioma (38, 39). However, IDH mutation in AML and chondrosarcoma has been linked to worse prognosis, while the opposite is true for glioma (38). This phenomenon implied the complex role in gliomagenesis. On one hand, IDH mutation may inhibit complement activation and help the tumor evade immune surveillance, leading to gliomagenesis (40, 41). On the other hand, IDH mutation reduced cytoprotection and apoptosis resistance in tumor cells and increased the number of antitumor immune cells (M1 tumor-associated macrophages), resulting in tumor suppression (42, 43). And the better prognosis of IDH-mu glioma patients implied that the tumor suppression role overwhelmed its promotion role, which led to a longer disease course and allowed contralesional reorganization to proceed as observed in the present study.

Moreover, opposite to the IDH subtype, we did not observe a compensation in the contralesional insular cortex in different histological grades. These results implied that the effect on contralesional compensation on the IDH subtype was more significant than histological classification, also provided evidence for different structural remodeling patterns in different IDH subtypes, and also supported the distinctive biological behavior between them (44). As for GBM, the most malignant type in histological classification could be divided into primary GBM (always IDH-wt) and secondary GBM (always IDH-mu). Our result implied the insular secondary GBM patients had a relatively long disease course, which is consistent with its feature of increasing the malignancy grade of a lower grade glioma over time (45).

There were some limitations to this study. Firstly, the sample in this study was relatively small. Secondly, we did not evaluate neurocognitive performance in our patients, and therefore, it is unclear whether the observed reorganization of the brain structure had functional significance. And functional MRI could provide additional information on this point. Additionally, we did not determine whether an association between molecular pathologic subtype in glioma and contralesional structural reorganization exists in tumors located outside the insula, although this warrants further study. Nonetheless, our results provide evidence for contralesional plasticity in the brain of patients with insular glioma and a possible molecular basis for this observation. These findings may have clinical significance in that insular glioma patients with IDH mutation may be candidates for more complete resection when an intraoperative pathologic diagnosis is available.

## Data Availability Statement

The raw data supporting the conclusions of this article will be made available by the authors, without undue reservation.

## Ethics Statement

The studies involving human participants were reviewed and approved by the Institutional Review Board of the Beijing Tiantan Hospital. Written informed consent to participate in this study was provided by the participants' legal guardian/next of kin.

## Author Contributions

ZHu designed the study, analyzed the data, and drafted the manuscript. JX provided the clinical and imaging data. GL and ZL performed the literature search. SS, YZ, ZHo, and JX reviewed and edited the manuscript. All authors contributed to the article and approved the submitted version.

## Conflict of Interest

The authors declare that the research was conducted in the absence of any commercial or financial relationships that could be construed as a potential conflict of interest.

## References

[1] Raz N Lindenberger U. Life-span plasticity of the brain and cognition: from questions to evidence and back. Neurosci Biobehav Rev. (2013) 37(9 Pt B):2195–200. 10.1016/j.neubiorev.2013.10.003 24140011

[2] Ismail FY Fatemi A Johnston MV. Cerebral plasticity: Windows of opportunity in the developing brain. Eur J Paediatr Neurol. (2017) 21:23–48. 10.1016/j.ejpn.2016.07.007 27567276

[3] Yuan T Zuo Z Ying J Jin L Kang J Gui S. Structural and functional alterations in the contralesional medial temporal lobe in glioma patients. Front Neurosci. (2020) 14:10. 10.3389/fnins.2020.00010 32153348 PMC7044242

[4] Cao Y D'Olhaberriague L Vikingstad EM Levine SR Welch KMA. Pilot Study of functional MRI to assess cerebral activation of motor function after poststroke hemiparesis. Stroke. (1998) 29:112–22. 9445338 10.1161/01.str.29.1.112

[5] Pineiro R Pendlebury S Johansen-Berg H Matthews PM. Functional MRI detects posterior shifts in primary sensorimotor cortex activation after stroke: evidence of local adaptive reorganization? Stroke. (2001) 32:1134–9. 10.1161/01.str.32.5.1134 11340222

[6] Fisicaro RA Jost E Shaw K Brennan NP Peck KK Holodny AI. Cortical plasticity in the setting of brain tumors. Top Magn Reson Imaging. (2016) 25:25–30. 10.1097/rmr.0000000000000077 26848558 PMC4970642

[7] Almairac F Duffau H Herbet G. Contralesional macrostructural plasticity of the insular cortex in patients with glioma: a VBM study. Neurology. (2018) 91:e1902–8. 10.1212/wnl.0000000000006517 30305447

[8] Han K Davis RA Chapman SB Krawczyk DC. Strategy-based reasoning training modulates cortical thickness and resting-state functional connectivity in adults with chronic traumatic brain injury. Brain Behav. (2017) 7:e00687. 10.1002/brb3.687 28523229 PMC5434192

[9] Xu J Elazab A Liang J Jia F Zheng H Wang W. Cortical and subcortical structural plasticity associated with the glioma volumes in patients with cerebral gliomas revealed by surface-based morphometry. Front Neurol. (2017) 8:266. 10.3389/fneur.2017.00266 28649229 PMC5465275

[10] Liu D Chen J Hu X Hu G Liu Y Yang K. Contralesional homotopic functional plasticity in patients with temporal glioma. J Neurosurg. (2020) 134:1–9. 10.3171/2019.11.Jns191982 31923896

[11] Duffau H Capelle L. Preferential brain locations of low-grade gliomas. Cancer. (2004) 100:2622–6. 10.1002/cncr.20297 15197805

[12] Li Z Li G Liu Z Pan Y Hou Z Wu L. Transcortical approach for insular gliomas: a series of 253 patients. J Neuro Oncol. (2020) 147:59–66. 10.1007/s11060-020-03390-2 32006193

[13] Gogolla N. The insular cortex. Curr Biol. (2017) 27:R580–6. 10.1016/j.cub.2017.05.010 28633023

[14] Wu AS Witgert ME Lang FF Xiao L Bekele BN Meyers CA. Neurocognitive function before and after surgery for insular gliomas. J Neurosurg. (2011) 115:1115–25. 10.3171/2011.8.Jns11488 21905800

[15] Herbet G Maheu M Costi E Lafargue G Duffau H. Mapping neuroplastic potential in brain-damaged patients. Brain. (2016) 139(Pt 3):829–44. 10.1093/brain/awv394 26912646

[16] Eckel-Passow JE Lachance DH Molinaro AM Walsh KM Decker PA Sicotte H. Glioma groups based on 1p/19q, IDH, and TERT promoter mutations in tumors. N Engl J Med. (2015) 372:2499–508. 10.1056/NEJMoa1407279 26061753 PMC4489704

[17] Herveyjumper SL Berger MS. Insular glioma surgery: an evolution of thought and practice. J Neurosurg. (2019) 130:9–16. 10.3171/2018.10.JNS181519 30611160

[18] Carabenciov ID Buckner JC. Controversies in the therapy of low-grade gliomas. Curr Treat Options Oncol. (2019) 20:25. 10.1007/s11864-019-0625-6 30874903

[19] Patel M Vogelbaum MA Barnett GH Jalali R Ahluwalia MS. Molecular targeted therapy in recurrent glioblastoma: current challenges and future directions. Expert Opin Investig Drugs. (2012) 21:1247–66. 10.1517/13543784.2012.703177 22731981

[20] Hegi ME Diserens AC Gorlia T Hamou MF de Tribolet N Weller M. MGMT gene silencing and benefit from temozolomide in glioblastoma. N Engl J Med. (2005) 352:997–1003. 10.1056/NEJMoa043331 15758010

[21] Ocklenburg S Friedrich P Güntürkün O Genç E. Voxel-wise grey matter asymmetry analysis in left- and right-handers. Neurosci Lett. (2016) 633:210–4. 10.1016/j.neulet.2016.09.046 27687715

[22] Yuan T Ying J Zuo Z Gui S Gao Z Li G. Structural plasticity of the bilateral hippocampus in glioma patients. Aging. (2020) 12:10259–74. 10.18632/aging.103212 32507763 PMC7346025

[23] Hartmann C Meyer J Balss J Capper D Mueller W Christians A. Type and frequency of IDH1 and IDH2 mutations are related to astrocytic and oligodendroglial differentiation and age: a study of 1,010 diffuse gliomas. Acta Neuropathol. (2009) 118:469–74. 10.1007/s00401-009-0561-9 19554337

[24] Fjell AM Westlye LT Grydeland H Amlien I Espeseth T Reinvang I. Accelerating cortical thinning: unique to dementia or universal in aging? Cereb Cortex. (2014) 24:919–34. 10.1093/cercor/bhs379 23236213 PMC3948495

[25] Storsve AB Fjell AM Tamnes CK Westlye LT Overbye K Aasland HW. Differential longitudinal changes in cortical thickness, surface area and volume across the adult life span: regions of accelerating and decelerating change. J Neurosci. (2014) 34:8488–98. 10.1523/jneurosci.0391-14.2014 24948804 PMC6608217

[26] Hedman AM van Haren NEM Schnack HG Kahn RS Hulshoff Pol HE. Human brain changes across the life span: a review of 56 longitudinal magnetic resonance imaging studies. Hum Brain Mapp. (2012) 33:1987–2002. 10.1002/hbm.21334 21915942 PMC6870052

[27] Battaglini M Gentile G Luchetti L Giorgio A Vrenken H Barkhof F. Lifespan normative data on rates of brain volume changes. Neurobiol Aging. (2019) 81:30–7. 10.1016/j.neurobiolaging.2019.05.010 31207467

[28] Ridgway GR Litvak V Flandin G Friston KJ Penny WD. The problem of low variance voxels in statistical parametric mapping; a new hat avoids a ‘haircut’. NeuroImage. (2012) 59:2131–41. 10.1016/j.neuroimage.2011.10.027 22037420 PMC3361668

[29] Di Pino G Pellegrino G Assenza G Capone F Ferreri F Formica D. Modulation of brain plasticity in stroke: a novel model for neurorehabilitation. Nat Rev Neurol. (2014) 10:597–608. 10.1038/nrneurol.2014.162 25201238

[30] Takeuchi N Tada T Chuma T Matsuo Y Ikoma K. Disinhibition of the premotor cortex contributes to a maladaptive change in the affected hand after stroke. Stroke. (2007) 38:1551–6. 10.1161/STROKEAHA.106.470187 17363726

[31] Shimizu T Hosaki A Hino T Sato M Komori T Hirai S. Motor cortical disinhibition in the unaffected hemisphere after unilateral cortical stroke. Brain. (2002) 125:1896–907. 10.1093/brain/awf183 12135979

[32] Lotze M Markert J Sauseng P Hoppe J Plewnia C Gerloff C. The role of multiple contralesional motor areas for complex hand movements after internal capsular lesion. J Neurosci. (2006) 26:6096–102. 10.1523/jneurosci.4564-05.2006 16738254 PMC6675223

[33] Heynen AJ Yoon BJ Liu CH Chung HJ Huganir RL Bear MF. Molecular mechanism for loss of visual cortical responsiveness following brief monocular deprivation. Nat Neurosci. (2003) 6:854–62. 10.1038/nn1100 12886226

[34] Butz M W?Rg?Tter F Ooyen AV. Activity-dependent structural plasticity. Brain Res Rev. (2009) 60:287–305. 10.1016/j.brainresrev.2008.12.023 19162072

[35] Draganski B May A. Training-induced structural changes in the adult human brain. Behaviou Brain Res. (2008) 192:137–42. 10.1016/j.bbr.2008.02.015 18378330

[36] Stroemer RP Kent TA Hulsebosch CE. Neocortical neural sprouting, synaptogenesis, and behavioral recovery after neocortical infarction in rats. Stroke. (1995) 26:2135–44. 7482662 10.1161/01.str.26.11.2135

[37] Ohba S Hirose Y. Biological significance of mutant isocitrate dehydrogenase 1 and 2 in gliomagenesis. Neurol Med Chir. (2016) 56:170–9. 10.2176/nmc.ra.2015-0322 26960449 PMC4831942

[38] Molenaar RJ Maciejewski JP Wilmink JW van Noorden CJF. Wild-type and mutated IDH1/2 enzymes and therapy responses. Oncogene. (2018) 37:1949–60. 10.1038/s41388-017-0077-z 29367755 PMC5895605

[39] Koivunen P Lee S Duncan CG Lopez G Lu G Ramkissoon S. Transformation by the (R)-enantiomer of 2-hydroxyglutarate linked to EGLN activation. Nature. (2012) 483:484–8. 10.1038/nature10898 22343896 PMC3656605

[40] Zhang L Sorensen MD Kristensen BW Reifenberger G McIntyre TM Lin F. D-2-Hydroxyglutarate is an intercellular mediator in IDH-mutant gliomas inhibiting complement and T cells. Clin Cancer Res. (2018) 24:5381–91. 10.1158/1078-0432.Ccr-17-3855 30006485 PMC6214730

[41] Philip B Yu DX Silvis MR Shin CH Robinson JP Robinson GL. Mutant IDH1 promotes glioma formation *in vivo*. Cell Rep. (2018) 23:1553–64. 10.1016/j.celrep.2018.03.133 29719265 PMC6032974

[42] Houillier C Wang X Kaloshi G Mokhtari K Guillevin R Laffaire J. IDH1 or IDH2 mutations predict longer survival and response to temozolomide in low-grade gliomas. Neurology. (2010) 75:1560–6. 10.1212/WNL.0b013e3181f96282 20975057

[43] Liu PS Wang H Li X Chao T Teav T Christen S. α-ketoglutarate orchestrates macrophage activation through metabolic and epigenetic reprogramming. Nat Immunol. (2017) 18:985–94. 10.1038/ni.3796 28714978

[44] Sanson M Marie Y Paris S Idbaih A Laffaire J Ducray F. Isocitrate Dehydrogenase 1 codon 132 mutation is an important prognostic biomarker in gliomas. J Clin Oncol. (2009) 27:4150–4. 10.1200/JCO.2009.21.9832 19636000

[45] Aldape K Zadeh G Mansouri S Reifenberger G von Deimling A. Glioblastoma: pathology, molecular mechanisms and markers. Acta Neuropathol. (2015) 129:829–48. 10.1007/s00401-015-1432-1 25943888

